# Size Effect of Stem Cell Spheroids in Their Cryopreservation Using Low-Molecular-Weight PEGs as a Cryoprotectant

**DOI:** 10.34133/bmr.0302

**Published:** 2026-01-06

**Authors:** Madhumita Patel, Solji Sung, Brent Vernon, Byeongmoon Jeong

**Affiliations:** ^1^ Department of Chemistry and Nanoscience, Graduate Program in Innovative Biomaterials Convergence, Ewha Womans University, Seoul 03760, Republic of Korea.; ^2^ Nebraska Translational Research Center (NTRC), Department of Growth and Development, College of Dentistry, University of Nebraska Medical Center, Omaha, NE 68105, USA.; ^3^ School of Biological and Health Systems Engineering, Arizona State University, Tempe, AZ 85287-9709, USA.

## Abstract

Cryopreservation is a crucial procedure to maintain quality of stem cell spheroids (SCSs) for a long period. It is affected by spheroid size because only internalized cryoprotectants can effectively play their roles within SCSs. Here, small, medium, and large SCSs with diameters of 30 to 80, 80 to 150, and 100 to 200 μm, respectively, were prepared using tonsil-derived stem cells. SCSs were preincubated in the presence of poly(ethylene glycol)s (PEGs) (10.0 wt % in a medium) with molecular weights of 200, 400, or 600 Da at 37 °C for 2 h, and then cryopreserved at −196 °C for 7 d. SCS recovery rate from cryopreservation was significantly affected by their size as well as molecular weight of PEGs; excellent recovery was observed for the small SCSs that were preincubated in PEG200 solutions. Population density of PEGs in the SCSs was 2.0 to 4.5 times higher in small SCSs than in large SCSs, which contributed to the survival of the SCSs during cryopreservation. The small SCSs recovered from cryopreservation showed innate activities of stem cells including fusion, proliferation, and differentiation much better than medium or large SCSs. The small SCSs employing the PEG200 preincubation protocol exhibited a cell recovery rate of >60% for 1 month of cryopreservation. These findings provide valuable insights into size-dependent cryopreservation strategies for high-level complex cellular systems.

## Introduction

Cryopreservation has significantly advanced biotechnologies by enabling the long-term storage of cells, tissues, and organs while preserving their biofunctions [1]. Dimethyl sulfoxide (DMSO) is the most frequently utilized cryoprotectant. Usually, culture medium containing 10% DMSO is employed to preserve cells. DMSO replenishes intracellular water and dries the cells, and lowers osmotic stress [2,3]. However, DMSO is cytotoxic and causes harmful consequences like histone acetylation, global DNA methylation, membrane disintegration, and protein denaturation [4,5]. Additionally, stem cell differentiation may be affected by DMSO [6]. Recently, promising cryoprotectants have been extensively searched based on the cryoprotection mechanisms of ice recrystallization inhibition (IRI) and reduction of osmotic stress of cells during freezing and thawing. Antifreezing protein-mimicked polypeptides, oligonucleotides, oligopeptides, dendritic polyglycerol, graphene oxides, and synthetic polymers such as poly(vinyl alcohol), amphipathic polymers, and poly(ethylene glycol) (PEG)-polypeptides are typical examples of cryoprotectants developed so far [3,4,7–14]. However, most of them were partially successful in achieving IRI activity, osmotic stress reducing activity, stability, ease of production, cyto- and bio-compatibility, and biodegradability, which might be required as an ideal cryoprotectant. In addition, the application of cryoprotectants can be cell-specific, thus a cryoprotectant working for some cells, but not working for other cells due to the differences in material–cell interactions. Therefore, still more research studies are required to develop specific cryoprotection systems.

As the demand for 3-dimensional (3D) cell culture models continues to grow in fields such as drug discovery, tissue engineering, and regenerative medicine, an effective cryopreservation method for these complex structures has become increasingly important [15,16]. A stem cell spheroid (SCS) is a sophisticated 3D model that closely replicates the native tissue microenvironments. SCSs promote essential cell–cell interactions, simulate nutrient gradients through the cellular aggregates, and exhibit physiologically relevant cellular behaviors better than 2D culture systems [16,17]. Nevertheless, cryopreserving these spheroids poses serious challenges to survival rate and recovery of biofunctions after cryopreservation, primarily due to their structural complexity and size-dependent issues. One of the main obstacles in spheroid cryopreservation is to ensure penetration of cryoprotectants into the cell aggregates. Larger spheroids are susceptible to cryoinjury due to poor cryoprotectant diffusion into the SCSs, and thus suffer from insufficient inhibition of intracellular/extracellular ice formation and development of osmotic pressure during cryopreservation. Therefore, structural integrity of cells comprising SCSs is disrupted and lead to significant cell death [18,19]. Furthermore, the nature of cryoprotectants is also important in minimizing osmotic stress and inhibiting ice crystal growth during freezing and thawing [20]. Recently, alternatives like polyampholytes and pollen-washing water were reported for spheroid cryopreservation; however, these are not self-sufficient and used DMSO as a co-cryoprotectant [19,21].

PEGs have gained attention for their ability to modulate ice formation and improve post-thaw recovery in various cell types. They have been used to successfully cryopreserve bacteriophages, microorganisms, and individual cells [22–24]. When combined with DMSO during cryopreservation, PEGs increased the survival rates of mouse and pig oocytes [25]. PEG coacervates have recently been reported to be a potent cryoprotectant for stem cells [26]. The exact mechanism by which PEGs work during freezing is yet unknown. However, it is reported that extracellular PEGs promote cell dehydration by generating osmotic pressure, which lowers intracellular ice formation and helps avoid cryo-injuries [27]. Additionally, PEGs prevent lipid peroxidation and stabilize cell membranes during sub-zero preservation [28,29]. In our previous studies, we proved that the molecular weight of PEGs significantly affected their effectiveness as cryoprotectants [23,24]. By diffusing into cells, low-molecular-weight PEGs efficiently inhibited ice recrystallization and improved cryoprotection for stem cells at the single-cell and spheroid level [24,30]. We examined the cryoprotective properties of PEGs with molecular weights of 200, 400, 600, 1,000, 1,500, 10,000, and 20,000 Da for both stem cells and SCSs of approximately 100 μm in size. We also evaluated the effect of preincubation at 37 °C for 0 h (no preincubation), 2 h, 4 h, and 6 h, and identified 2 h as the optimal preincubation time for enhancing post-thaw recovery of SCSs. Furthermore, we demonstrated that low-molecular-weight PEGs (<1,000 Da) entered cells via passive diffusion, whereas high-molecular-weight PEGs (20 kDa) were internalized through clathrin-mediated endocytosis, which was inhibited by chlorpromazine [24,30]. Despite these findings, the effect of low-molecular-weight PEGs on SCS cryopreservation, particularly with respect to spheroid size, has not been investigated.

In this study, small, medium, and large SCSs (30 to 80, 80 to 150, and 100 to 200 μm in diameter, respectively) were prepared using tonsil-derived stem cells. We then examined the effects of PEGs with low molecular weights of 200, 400, and 600 Da (coded as PEG200, PEG400, and PEG600 in this paper) on the cryopreservation of these SCSs. Tonsil-derived mesenchymal stem cells (MSCs) are known to exhibit higher proliferative capacity and shorter doubling times compared with bone marrow-derived MSCs. They possess multipotent differentiation capability into adipogenic, chondrogenic, osteogenic, and hepatogenic lineages [31]. The recovered SCSs from cryopreservation at −196 °C for 7 d were also checked for their healthy states by biofunctions of SCSs such as fusibility, proliferation, and potentials for adipogenic, chondrogenic, and osteogenic differentiation. In addition, cell recovery rate from 1-month cryopreservation at −196 °C was also studied for small SCSs using PEG200.

## Materials and Methods

### Materials

PEGs were used as supplied (molecular weights of 200 and 400 Da: Tokyo Chemical Industry Co. Ltd., Japan; 600 Da: Sigma-Aldrich, USA). Dulbecco’s modified Eagle’s medium (DMEM) and its supplements including streptomycin, penicillin, and fetal bovine serum (FBS) were used as received from Corning, USA.

### Stem cell culture and spheroid formation

Tonsil-derived MSCs used in this work were generously donated by the Ewha Womans University Medical School following Institutional guidelines. The cells were cultivated in a sterile incubator set at 37 °C under 5% CO_2_ conditions in DMEM supplemented with 10% (v/v) FBS, 1% (v/v) antibiotic–antimitotic, and 1% (v/v) penicillin/streptomycin using cell culture plate (Corning 150-mm tissue culture-treated Culture Dish, Corning, USA). The cells were used for spheroid preparation after being cultivated until passage 6. Trypsin was used to collect confluent cells. The stem cell suspension (0.8 ml) at densities of 0.1 × 10^6^, 1.0 × 10^6^, and 1.5 × 10^6^ cells/ml in DMEM solutions was added to spheroid dish (SPL3D, SPL Life Science, Republic of Korea) made of polystyrene dish/polyethylene mesh with internal well dimension of 25 mm × 1 mm and mesh pore size of 200 μm to prepare small (30 to 80 μm), medium (80 to 150 μm) and large (100 to 200 μm) SCSs, respectively. The plates were kept in an incubator at 37 °C for 24 h. Spheroids with different sizes were collected in phosphate-buffered saline (PBS) and utilized for the cryopreservation study.

### Cryopreservation and cell recovery

Cryovials (Thermo Scientific Nunc, USA) containing 0.5 ml of a specific PEG solution (10.0 wt % in DMEM) have been prepared. These cryovials were used to hold approximately 300 SCSs of different sizes per vial. These vials were then incubated at 37 °C under 5% CO_2_ conditions for 0 h (no incubation) and 2 h. Then, the cryopreservation procedure followed a slow cooling protocol previously reported [16,32,33]. Briefly, the vials were transferred to a cryo box (Mr. Frosty; Thermo Scientific) to −80 °C in a controlled freezing container. After a 12-h incubation, the cryovials were transferred to liquid nitrogen (−196 °C) and stored for 7 d. The cryovials were then removed from the liquid nitrogen and thawed in a water bath set at 37 °C. The spheroids were moved to a conical tube and then diluted 10 times with DMEM growth media. The spheroids were then centrifuged at 1,500 rpm for 5 min, and the supernatant was then removed. Then, the SCSs were resuspended in 0.5 ml of growth medium. The viability of the spheroids was then assessed using a cell counting kit-8 (CCK-8; Dojindo, Japan) and a live/dead test kit (Invitrogen, USA). In addition, we compared the recovery rates of SCSs after 1 month of cryopreservation at −196 °C among 3 conditions: DMEM alone,10% PEG200 in DMEM, and 10% DMSO in DMEM, following the protocol described above. The recovered SCSs were subsequently evaluated using live/dead and the CCK-8 assay.

### Cytotoxicity of low-molecular-weight PEGs

The cytotoxicity of PEG200, PEG400, and PEG600 at 10.0 wt % in DMEM was evaluated for spheroids in suspension after incubating SCSs for 0 and 2 h at 37 °C under 5% CO_2_ conditions [24,30]. Then, SCSs were assayed using live/dead and CCK-8 assay according to the manufacturer’s protocols.

### PEG internalization

Internalization of PEGs into SCSs was studied by a similar procedure published elsewhere [30,34]. Briefly, about 300 SCSs were suspended in a DMEM solution (0.5 ml) in the presence of PEG200, PEG400, or PEG600 at a concentration of 100 μM and incubated for 2 h at 37 °C. Then, solutions were centrifuged for 5 min at 1500 rpm to collect the SCSs. They were resuspended in PBS (0.5 ml) and centrifuged. The same procedure was repeated 3 times to remove PEGs outside of the SCSs. The spheroids were then resuspended in 0.4 ml of PBS and processed ultrasonically for 1 min at 30 amps using an ultrasonic processor manufactured by Sonics and Materials Inc. (USA). After ultrasonication, the samples were centrifuged for 20 min at 9,000*g* to eliminate cellular debris, and the resulting supernatant was collected. The levels of intracellular PEG extracted from the supernatant were quantified using an enzyme-linked immunosorbent assay (ELISA) kit for PEGs from MyBioSource, USA.

### Computer simulation on PEG internalization into SCSs

To evaluate the effect of spheroid size on PEG internalization, the diffusion of PEG200, PEG400, and PEG600 into SCSs with diameters of 55 μm (small), 115 μm (medium), and 150 μm (large) was modeled using Fick’s second law of diffusion in spherical coordinates [35]. The dimensionless diffusion equation is:

$$\frac{\partial C^{*}}{\partial t^{*}}=\frac{D^{*}}{r^{*2}}\frac{\partial }{\partial r^{*}}\left(r^{*2}\frac{\partial C^{*}}{\partial r^{*}}\right)$$ (1)

where C^*^ = *C*/*C*_0_ is the normalized concentration (*C*_0_ = 1, dimensionless external concentration), r* = *r*/*R* is the normalized radial position (*R* is the physical radius: 27.5, 57.5, and 75 μm), t* = *tD*/*R*^2^ is the nondimensional time, and D* = *D*/*D*_0_ is the relative diffusion coefficient (*D*_0_ = 0.01 μm^2^/s for PEG200). Boundary conditions were C* = 1 at r* = 1 (Dirichlet) and $\frac{\partial C^{*}}{\partial r^{*}}=0$ at r* = 0 (Neumann, symmetry). Initial conditions set C* = 0 at t* = 0.

Diffusion coefficients were scaled as $D^{*}\propto M_{w}^{-0.43}$ based on Shimada et al. [36], yielding D* = 1.0, 0.740, and 0.624 for PEG200, PEG400, and PEG600, respectively (*D* = 0.01, 0.0074, and 0.00624 μm^2^/s). Physical time was computed using *t* = t*R^2^/(D*D_0_).

The equation was solved numerically in Python using an explicit finite difference method with 100 radial points (Δr* = 0.01) and 200,000 time steps (Δt* = 5 × 10^−5^) over a nondimensional time T* = 10.0, ensuring stability via the Courant–Friedrichs–Lewy condition (D*Δt*/Δr* ≤ 0.5). At the spheroid center (r* = 0), the update was as follows:

$$\begin{array}{c} C^{*}\left(0,t^{*}+∆t^{*}\right)=C^{*}\left(0,t^{*}\right)+6∆t^{*}D^{*}\frac{C^{*}\left(r_{1}^{*},t^{*}\right)-C^{*}\left(0,t^{*}\right)}{∆r^{*2}} \end{array}$$ (2)

The uptake was calculated as:

$$\frac{m_{t}}{m_{\infty }}=\frac{3}{C_{0}}\int_{0}^{1}C^{*}\left(r_{1}^{*},t^{*}\right)r^{*2}dr^{*}$$ (3)

Simulations were performed for all combinations of PEG molecular weights and SCS sizes.

### Fusibility of recovered SCSs

The recovered SCSs from cryopreservation at −196 °C for 7 d were assessed for their ability to undergo spheroid fusion. They were seeded into 96-well ultra-low attachment plates (Nunclon Sphera 96-Well Microplate, Thermo Fisher Scientific, USA) and cultured for 3 d in growth media. Images of the spheroid fusion were captured using an Olympus IX71 microscope with Olympus DP2-BSW software.

### Proliferation of recovered SCSs

In order to investigate cell proliferation, the recovered SCSs from cryopreservation at −196 °C for 7 d were seeded into a 96-well plate (Nunc Micro Well 96-Well Microplate, Thermo Fisher Scientific, USA). After 3 d of growth, cell images were captured using a live/dead test kit. The cells were treated with a solution containing calcein AM (2.0 μM) and ethidium homodimer-1 (2.0 μM) for 30 min. Fluorescence images were then captured using an Olympus IX71 microscope in conjunction with Olympus DP2-BSW software. To quantify cell proliferation, the growth medium was replaced with CCK-8 solution (10% in DMEM supplemented with 1% penicillin/streptomycin), and this mixture was incubated for 150 min at 37 °C. Stem cell proliferation was also assessed semiquantitatively by measuring absorbance at 450 nm, with the data collected at day 0 (3 h) serving as a reference value of 100% for the assay.

### Stem cell differentiation

Differentiation potential of retrieved SCSs was assessed for their capacity to develop into osteogenic, chondrogenic, and adipogenic lineages. SCSs were seeded on a 24-well plate (Nunc Cell-Culture Treated Multidishes, Thermo Fisher Scientific, USA), and the media were refreshed every 2 d. Upon reaching confluence, the cells were treated for 2 weeks with specific induction media for osteogenic, chondrogenic, and adipogenic differentiation, with media changes occurring every 2 d [33,37]. After fixation with 4% formaldehyde and rinsing with PBS, the cells were stained with Alizarin Red, Alcian Blue, and Oil Red O to visualize osteogenic, chondrogenic, and adipogenic differentiation, respectively. Images were captured using an Olympus IX71 fluorescent microscope in conjunction with Olympus DP2-BSW software. The images were semiquantitatively analyzed using ImageJ software, and the percentage of the stained area was expressed. The recovered SCSs obtained from cryopreservation using the conventional 10% DMSO method were also evaluated as a control. In addition, mRNA expression levels associated with osteogenic, chondrogenic, and adipogenic differentiation were analyzed using runt-related transcription factor 2 (RUNX2), type II collagen (COL II), and peroxisome proliferator-activated receptor γ (PPAR), respectively, following the same protocol previously published [37].

### Statistical analysis

Experiments were conducted in triplicate for statistical data treatments. Tukey tests and a one-way analysis of variance (ANOVA) were used to assess the data’s significance. *P* values less than 0.05 and 0.01 were used to indicate statistical significance, and these differences were denoted by the symbols * and **, respectively.

## Results and Discussion

### Preparation of SCSs

**Fig. 1.**
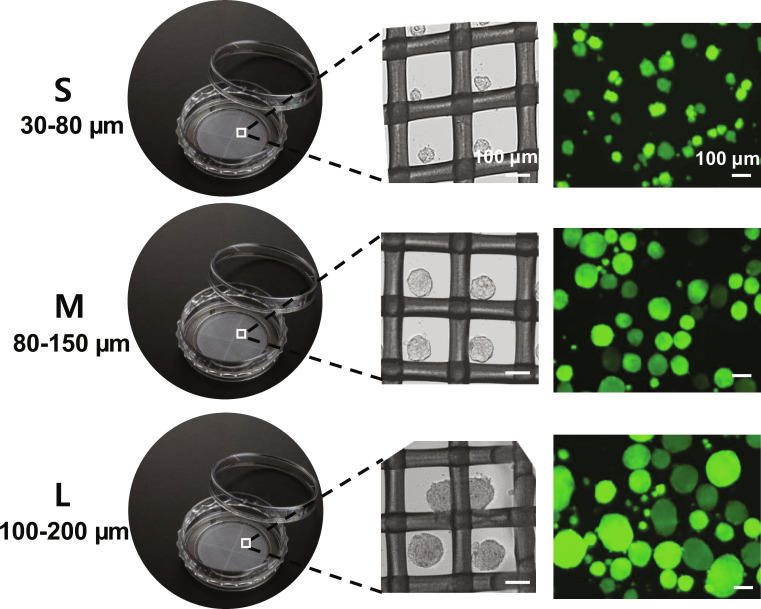
Preparation of stem cell spheroids (SCSs) with different sizes. Small (S: 30 to 80 μm), medium (M: 80 to 150 μm), and large (L: 100 to 200 μm) SCSs were prepared by adding DMEM solution (0.8 ml) containing 0.1 × 10^6^, 1.0 × 10^6^, and 1.5 × 10^6^ cells/ml in spheroid dish with a mesh pore size of 200 μm. The phase-contrast images of the SCSs in the wells (middle) are presented in black and white. Live (green) and dead (red) images of the finally collected SCSs after detachment from the wells (right) are also shown.

Small (30 to 80 μm), medium (80 to 150 μm), and large (100 to 200 μm) SCSs were prepared by varying cell numbers of 0.1 × 10^6^, 1.0 × 10^6^, and 1.5 × 10^6^ cells/ml, respectively, in the spheroid dish made of coated polystyrene and polyethylene mesh. The phase contrast images and live/dead images of the SCSs with different sizes are shown in Fig. 1.

### Cryopreservation of SCSs

**Fig. 2.**
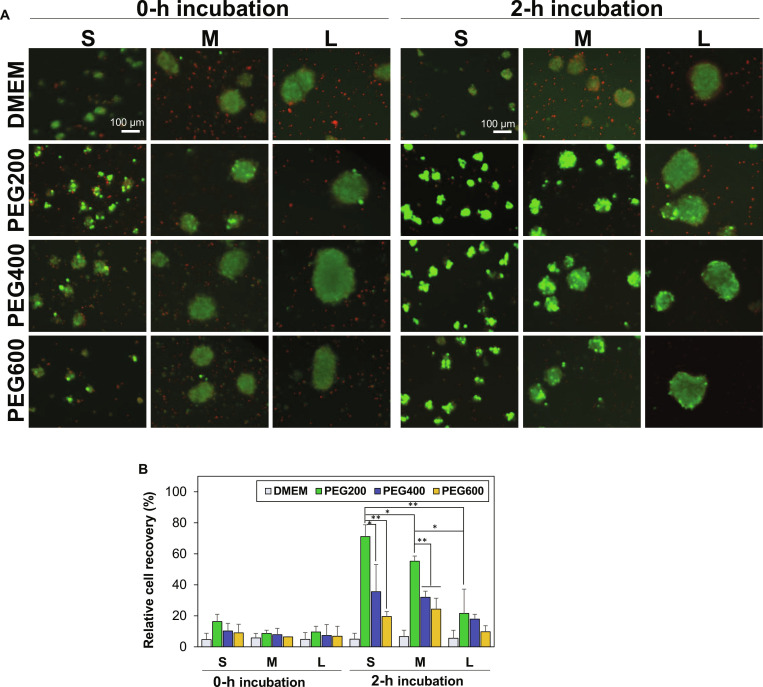
(A) Live and dead images of SCSs recovered from cryopreservation at −196 °C for 7 d. Before cryopreservation, SCSs with small (S), medium (M), and large (L) sizes were preincubated for 0 h (no incubation) or 2 h in the presence of PEG200, PEG400, and PEG600 at 10.0 wt % in DMEM at 37 °C. Scale bar, 100 μm. (B) Quantitative analysis for SCS recovery rate from cryopreservation assayed using the CCK-8 kit. Each group was triplicated (*N* = 3). *P < 0.05, **P < 0.01.

The SCSs were preincubated in PEG solutions (10.0 wt %) at 37 °C for 2 h before cryopreservation. We reported that 2-h preincubation of cells in the presence of low-molecular-weight PEGs significantly improve cell recovery rate from cryopreservation [24,30]. In this study, SCSs with different sizes were preincubated in the presence of PEG200, PEG400, and PEG600 at 10.0 wt %. Then, a typical slow freezing protocol was employed for cryopreservation of SCSs as described in Materials and Methods [16,32,33]. Many cells within the SCSs died during cryopreservation, as indicated in red in the live/dead images, when SCSs were preincubated in DMEM solution in the absence of PEGs or when the SCSs were directly subjected to cryopreservation without preincubation procedure (0-h incubation) (Fig. 2A). The 0-h preincubation indicates that SCSs suspended in PEG solutions (10.0 wt % in DMEM) were subjected to direct cryopreservation without preincubation at 37 °C. This study confirmed again the importance of 2-h preincubation of SCSs at 37 °C in the presence of low-molecular-weight PEGs. The preincubation in the presence of PEGs, particularly PEG200, significantly improved the cell recovery rate after cryopreservation of SCSs. The effectiveness of preincubation was in an order of PEG200 > PEG400 > PEG600. Quantitative analysis on the cell recovery rate showed that the cell recovery rate was very poor (<16%) when SCSs were preincubated in a DMEM solution in the absence of PEGs or when the SCSs were directly subjected to cryopreservation (0-h incubation) (Fig. 2B). In particular, the cell recovery rate for small SCSs, medium SCSs, and large SCSs exhibited 71%, 55%, and 22%, respectively in the presence of PEG200. This finding suggests that the size of SCSs is a critical factor in determining the cell recovery rate from cryopreservation. When the small SCSs were compared for the different molecular weight of PEGs used as a cryoprotectant, the cell recovery rate was found to drop from 71% to 36%, and to 20% as the PEG molecular weight increased from 200 to 400, and to 600, respectively. To conclude, the size of SCSs, as well as the PEG molecular weight, is a determining factor in cell recovery from cryopreservation.

**Fig. 3.**
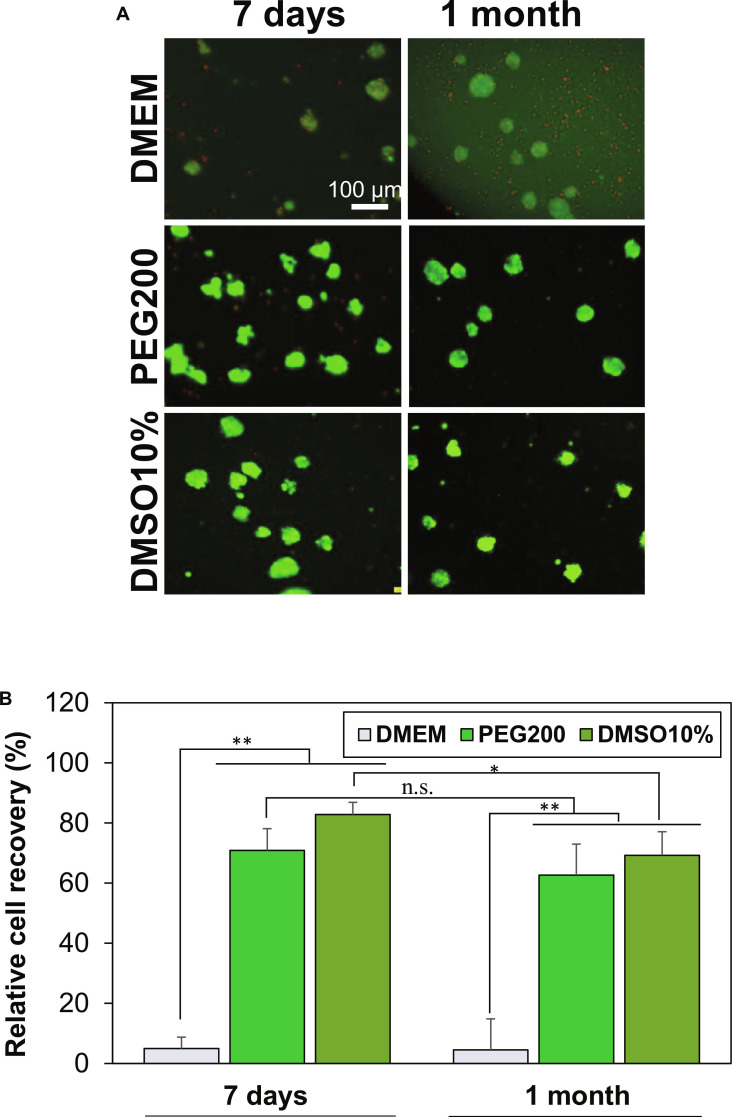
Cell recovery from long-term cryopreservation of small SCSs at −196 °C for 7 d and 1 month. (A) Representative live/dead fluorescence images recovered SCSs. DMEM: Small SCSs preincubated in DMEM at 37 °C for 2 h without PEGs. PEG200: Small SCSs preincubated in 10.0 wt % PEG200 in DMEM at 37 °C for 2 h prior to cryopreservation. DMSO10%: Small SCSs cryopreserved in 10.0% DMSO in DMEM (conventional method) without preincubation at 37 °C. Scale bar, 100 μm. (B) Quantitative analysis for SCS recovery rate from cryopreservation. Data are presented as mean ± SD (*N* = 3). *P < 0.05, **P < 0.01. n.s., not significant.

To evaluate long-term cryopreservation performance, small SCSs were stored at −196 °C for 1 month. Representative images of SCSs and recovery rates are shown in Fig. 3A and B. When SCSs were cryopreserved in DMEM alone for 7 d or 1 month, less than 5% of cells were recovered. In contrast, when SCSs were preincubated for 2 h in 10.0 wt % PEG200 in DMEM before cryopreservation, recovery rates increased markedly to 71% and 63% after 7 d and 1 month of cryopreservation, respectively. No significant difference was observed between the 2 time points, suggesting that recovery efficiency is primarily influenced by the cooling and reheating processes rather than storage duration. The traditional cryopreservation method employs 10% DMSO. However, tonsil-derived MSCs suspended at 37 °C in DMEM containing 10% DMSO showed ~90% cell death [14]. Therefore, SCSs in the 10% DMSO system were cryopreserved without the 2-h preincubation step. Using this protocol, cell recovery rates were 83% after 7 d and significantly decreased to 69% after 1 month of cryopreservation. These recovery rates are comparable to, or slightly lower than, previously reported results [38,39]. For example, human dermal fibroblasts maintained >80% viability after cryopreservation with 10% DMSO at −196 °C for 1 month [38]. Under ??GMP-like conditions using DMEM/FBS with 10% DMSO or a DMSO-free medium (CryoOx), >90% recovery of progenitor cells (FE002-SK2) was achieved after cryopreservation at −196 °C for 56 months [39]. As SCSs consist of densely aggregated cells, long-term cryopreservation remains challenging, and recovery rates still require improvement. Nevertheless, the 2-h preincubation in 10.0 wt % PEG200 developed in this study demonstrates a promising alternative protocol for effective spheroid cryopreservation.

### Mechanism of difference in cell recovery

**Fig. 4.**
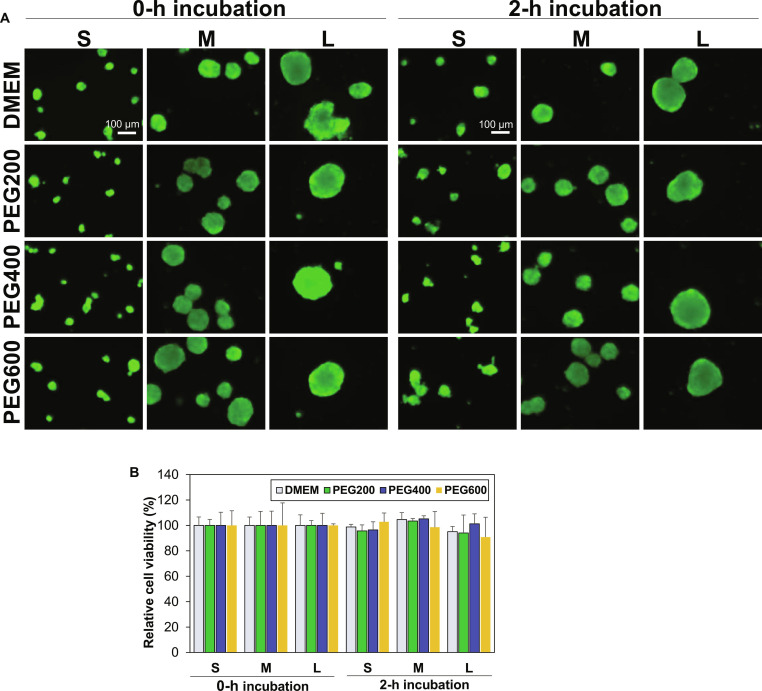
Cytotoxicity assay of PEGs for SCSs. (A) Live and dead images of SCSs in the presence of PEG200, PEG400, and PEG600 at 10.0 wt % in DMEM. The SCSs were suspended in the PEG solutions and incubated at 37°C for 0 h (no incubation) or 2 h. Scale bar, 100 μm. (B) Cell viability of SCSs assayed using the CCK-8 kit.

To understand the difference in cell recovery rate from cryopreservation, cytotoxicity of PEGs at 10.0 wt % was studied first because the SCSs might die at this high PEG concentration during 2-h preincubation at 37 °C. Live/dead images of SCSs with different sizes indicated that all SCSs were alive during the preincubation and they were stained in green (Fig. 4A). CCK-8 analyses of SCSs also confirmed that PEG200, PEG400, and PEG600 are cyto-compatible for all SCSs at 10.0 wt % concentration during the preincubation at 37 °C for 2 h (Fig. 4B).

**Fig. 5.**
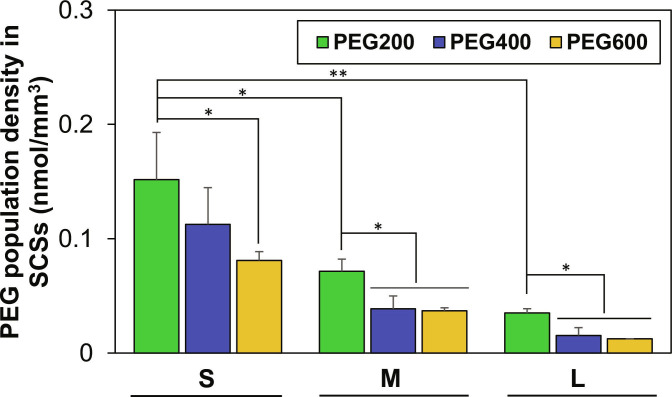
Internalization of PEGs into SCSs of different sizes. SCSs were incubated in PEG solutions (100 μM in DMEM) for 2 h. Each group was tested in triplicate (*N* = 3). Internalized PEG levels were quantified using a PEG-specific ELISA assay kit. *P < 0.05, **P < 0.01.

Second, the difference in internalization of PEGs might be responsible for the difference in cell recovery. The PEGs inside cells or cell spheroids can reduce recrystallization or nucleation of ice crystals and suppress osmotic pressure development during cryopreservation of cells or cell spheroids. The low-molecular-weight PEGs (<1,000 Da) are known to enter the cell by diffusion [40,41]. Diffusion of PEGs into SCSs and the resulting population density of PEGs in the SCSs can affect the cell recovery from cryopreservation. PEGs internalized into SCSs were quantified following 2-h preincubation at 37°C. The ELISA assay using PEG antibodies indicated that the population density of PEGs inside the SCSs decreased as the size of SCSs increased (Fig. 5). Population density of PEG200 in small SCSs (30 to 80 μm) was 4-fold higher than that of large SCSs (100 to 200 μm). PEG400 and PEG600 exhibited a similar trend with 3- to 7-fold higher population density in small SCSs than large SCSs. In addition, the lower molecular weight PEGs internalized into the SCSs better than larger molecular weight PEGs. PEG200 entered the SCSs 2-fold better than PEG600 for small, medium, and large SCSs. Based on above results, we can conclude that the high recovery of small SCSs using PEGs, particularly PEG200, comes from the easy diffusion of low-molecular-weight PEGs into small SCSs.

**Fig. 6.**
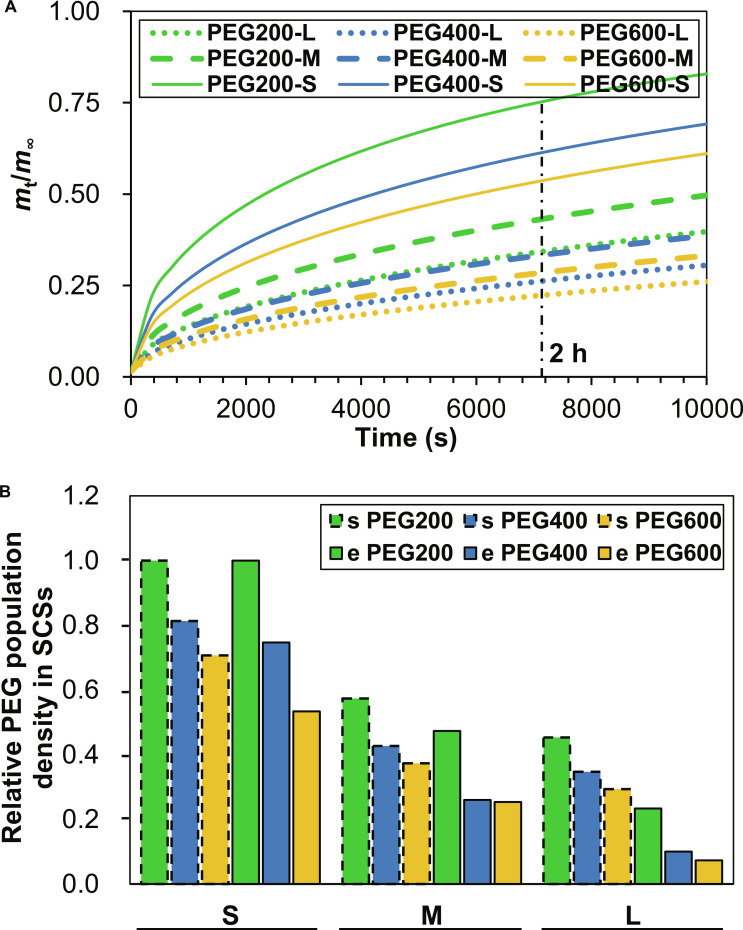
Simulation of PEG internalization into SCSs. (A) Relative uptake (*m*_t_/*m*_∞_) versus time for PEG200, PEG400, and PEG600 in 55-, 115-, and 150-μm SCSs. (B) Bar graph comparing simulated (s PEGs, presented as dotted bar graphs) and experimental (e PEGs) relative uptake trends at approximately 2 h, grouped by size.

To further investigate the size-dependent internalization of low-molecular-weight PEGs into SCSs, numerical simulations were performed for PEG200, PEG400, and PEG600 diffusion into SCSs with diameters of 55, 115, and 150 μm using Fick’s second law in spherical coordinates [35]. Diffusion coefficients were scaled as $D^{*}\propto M_{w}^{-0.43}$, per Shimada et al. [36], yielding *D* = 0.01, 0.0074, and 0.00624 μm^2^/s for PEG200, PEG400, and PEG600, respectively, with a normalized external concentration (*C*_0_ = 1). The apparent diffusion coefficient for PEG200, determined through iteration to find appropriate uptake ratios in the SCSs at 2 h, is significantly lower than the expected value, approximately 600 μm^2^/s in water per Shimada et al., suggesting a substantial reduction in diffusion within the cellular environment of SCSs. Figure 6A shows the relative uptake (*m*_t_/*m*_∞_) versus time for all 9 conditions (3 PEGs × 3 sizes). Smaller spheroids exhibited faster uptake kinetics due to shorter diffusion path lengths. For the 55-μm SCSs at 2 h, PEG200 reached an *m*_t_/*m*_∞_ of approximately 0.75, compared to ≈0.61 for PEG400 and ≈0.53 for PEG600. Larger spheroids showed a reduced uptake, with PEG200 achieving relative uptakes of approximately 0.43 and 0.34 for 115 and 150 μm SCSs, respectively. Lower molecular weight PEGs diffused more rapidly within each size due to higher diffusion coefficients. Figure 6B presents a bar graph comparing simulated and experimental relative uptake trends at approximately 2 h (7,200 s), grouped by spheroid size. Both datasets reveal a decline in uptake with increasing molecular weight and spheroid size, reflecting diffusion limitations. Simulated trends show a gradual reduction, while experimental data indicate a slightly steeper decline, particularly for higher molecular weight PEGs in larger spheroids. This suggests the model captures the overall size-dependent behavior but underestimates the extent of reduction observed experimentally. These results depend on the apparent diffusion coefficient (*D*_0_ = 0.01 μm^2^/s); smaller *D*_0_ values could enhance alignment with the observed size effect. The $M_{w}^{-0.43}$ scaling, derived for water, may not fully account for cellular barriers in SCSs, with alternative scalars potentially improving accuracy [36]. These findings support the experimental trend of reduced PEG uptake with increasing SCS size, aligning with cell recovery (Fig. 2B) and internalization data (Fig. 5). The superior uptake of PEG200 in 55-μm SCSs enhances cryoprotection by reducing intracellular ice formation and osmotic stress [24]. The model’s weaker size effect and higher uptake for PEG400 and PEG600 likely stem from the reduced apparent diffusion coefficient and $M_{w}^{-0.43}$ limitations. A possible improvement to the model would be to use a partition coefficient to represent diffusion of PEGs from the external medium into the extracellular space of the SCS, followed by a first-order rate constant to model internalization into the cells within the SCS. Future models incorporating the proposed improvements could refine predictions. However, these results still validate low-molecular-weight PEGs, especially PEG200, for optimizing cryopreservation of smaller SCSs in tissue engineering.

PEGs are generally regarded as safe (GRAS) and exhibit low toxicity, and consequently, they have been widely used in pharmaceutical formulation as inactive excipients [42]. In our previous studies, the mean largest grain size (MLGS) of 10.0 wt % aqueous PEG solution decreased from 68% to 40% relative to PBS as the molecular weight of PEG increased from 200 to 20,000 Da, indicating that IRI effects contribute only marginally to SCS stabilization during cryopreservation [23,24,30]. On the other hand, membrane stabilization effects of PEGs were demonstrated through measurements of calcein AM fluorescence decay in MSCs [23,28]. Trypan blue enters through destabilized cell membrane and quenches intracellular fluorescence; however, low-molecular-weight PEGs effectively stabilized the membrane, maintaining high fluorescence intensity, whereas DMSO markedly disrupted the cell membrane, resulting in significant fluorescence quenching [24,30]. Therefore, the cryoprotection mechanism of low-molecular-weight PEGs is attributed to their ability to stabilize the cell membrane while reducing osmotic stress and ice recrystallization through diffusion-based internalization into SCSs.

### Fusibility of the recovered SCSs

**Fig. 7.**
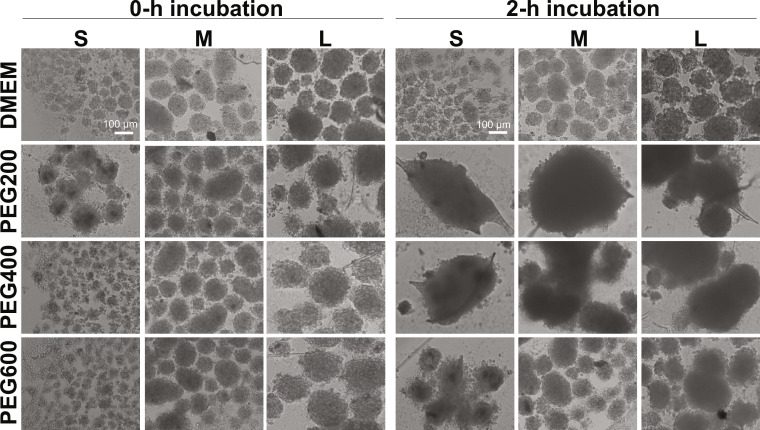
The fusibility of the recovered SCSs from cryopreservation at −196 °C for 7 d. The recovered SCSs were cultured on ultra-low attachment plates at 37 °C for 3 d and evaluated for fusibility of SCSs. Scale bar, 100 μm.

Health states of the recovered SCSs from cryopreservation are important for practical applications of the SCSs. In order to prove the health states, fusibility, proliferation, and differentiation potentials of SCSs were investigated. Tissue fusion is a basic mechanism for organ generation. Therefore, the fusibility of SCSs can be a measure of their health state [21,43–45]. The fusibility of the SCSs recovered from cryopreservation at −196 °C for 7 d was examined by seeding SCSs on ultra-low attachment plates. The excellent fusibility of the small and medium size SCSs was observed for the SCSs preincubated in the presence of PEG200 before cryopreservation (Fig. 7). The small SCSs preincubated in the presence of PEG400 also exhibited excellent fusibility. However, the SCSs cryopreserved without preincubation or preincubated in the absence of PEGs or in the presence of PEG600 did not exhibit good fusibility. This finding indicated that the size of SCSs, as well as the molecular weight of PEG used as a cryoprotectant, is a critical factor in maintaining the biofunctions of the recovered SCSs from cryopreservation.

### Proliferation of the recovered SCSs

**Fig. 8.**
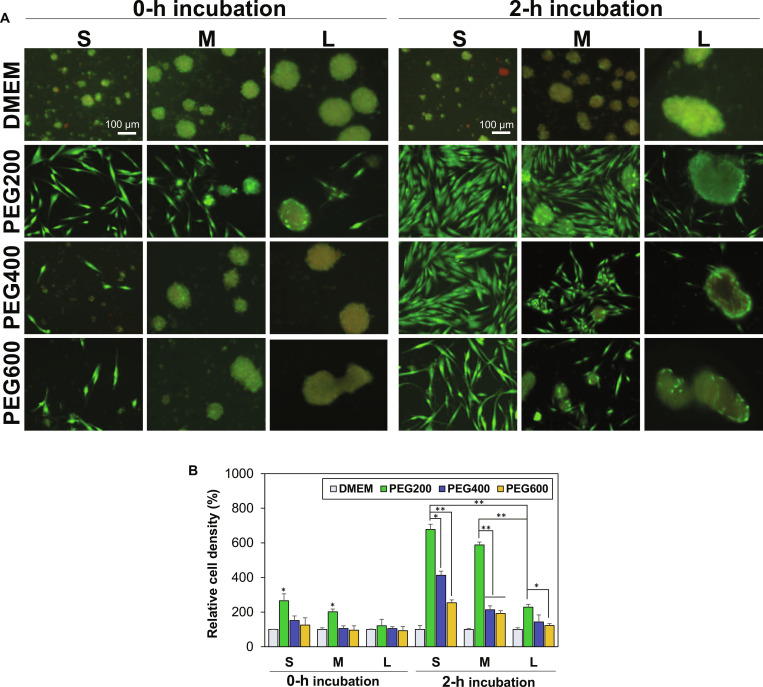
Proliferation of SCSs recovered from cryopreservation at −196 °C for 7 d. (A) Live/dead images of the cells taken 3 d after proliferation. Scale bar, 100 μm. (B) Quantitative analysis of cell proliferation relative to day 0 (100%), measured using the CCK-8 assay. Each group was studied in triplicate (*N* = 3). *P < 0.05, **P < 0.01.

Proliferation of SCSs recovered from cryopreservation was investigated to prove their healthy state. The recovered SCSs were cultured on the traditional polystyrene 2D plate. Regardless of SCS size, cell proliferation was unsuccessful for the SCSs subjected to direct cryopreservation without preincubation (0-h incubation) in low-molecular-weight PEGs (Fig. 8A). Similar to the SCS fusibility, excellent proliferation of the small and medium size SCSs was observed for the SCSs that had been preincubated for 2 h in the presence of PEG200 before cryopreservation. In addition, similar excellent results were observed for small SCSs that had been preincubated in the presence of PEG400. As the size of SCSs increased, the proliferation activity dropped significantly. In particular, large size SCSs recovered from cryopreservation exhibited a very low proliferation activity regardless of molecular weight of PEGs used for 2-h preincubation. Preincubation of SCSs in the presence of PEG400 and PEG600 was not effective to maintain the proliferation activity of the recovered SCSs with a medium size SCSs. Quantitative analysis indicated that proliferation activity of medium and large SCSs dropped to 87% and 34% of that of small SCSs, respectively, even for preincubation in the presence of PEG200 before cryopreservation (Fig. 8B). As for PEG molecular weight effect, the proliferation activity of small SCSs incubated in the presence of PEG400 and PEG600 dropped 61% and 37% of SCSs preincubated in the presence of PEG200, respectively, before cryopreservation. This finding also indicated that the size of SCSs, as well as the PEG molecular weight used in preincubation, must be a determining factor in maintaining the cell proliferation activity of SCSs after cryopreservation, similar to the above SCS fusibility results.

### Differentiation of the recovered SCSs

**Fig. 9.**
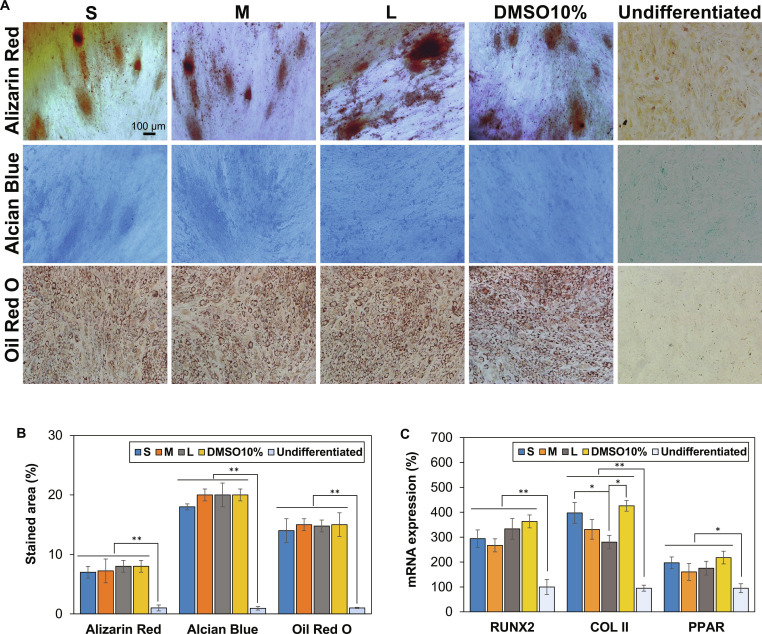
Differentiation potential of recovered SCSs following cryopreservation at −196 °C for 7 d. Small, medium, and large SCSs preincubated in 10.0 wt % PEG200-containing DMEM at 37 °C for 2 h prior to cryopreservation were evaluated. For comparison, SCSs cryopreserved using conventional 10% DMSO method were also examined. Undifferentiated stem cells were included as a negative control. (A) Representative images of osteogenic, chondrogenic, and adipogenic differentiation. Cells were stained with Alizarin Red (red-brown; osteogenic), Alcian Blue (blue; chondrogenic), and Oil Red O (red; adipogenic). (B) Semiquantitative analysis of staining intensities using ImageJ software. (C) mRNA expression of lineage-specific markers in differentiated cells.

Finally, the potentials for adipogenic, chondrogenic, and osteogenic differentiation were investigated for the SCSs recovered from cryopreservation. The SCSs preincubated at 37 °C 2 h in the presence of PEG200 were selected in this study to focus the size effect of SCSs. Oil Red O, Alcian Blue, and Alizarin Red stain the lipids and triglycerides of adipocytes, sulfated proteoglycans of chondrocytes, and calcium ions of osteocytes in red-brown, blue, and red, respectively [33,46,47]. Compared with the undifferentiated stem cells, the differentiated cells were deeply stained in brown, blue, and red, respectively (Fig. 9A). The images were also analyzed using ImageJ software, and the percentage of the stained area was semiquantitatively expressed. The cells subjected to adipogenic, chondrogenic, and osteogenic differentiation were significantly (*P* < 0.01) high stained than undifferentiated cells (Fig. 9B). These results demonstrate that SCSs recovered after cryopreservation with PEG200 or 10% DMSO method maintained robust differentiation capacity. Expression of lineage-specific markers—RUNX2 (osteogenic), COL II (chondrogenic), and PPAR (adipogenic)—was assessed at the mRNA level (Fig. 9C). Strong up-regulation of these genes verified that the recovered SCSs preserved their intrinsic multilineage differentiation potentials.

## Conclusion

SCSs of tonsil-derived MSCs with diameters of 30 to 80 μm (small), 80 to 150 μm (medium), and 100 to 200 μm (large) were prepared by varying the initial cell seeding numbers in spheroid dishes. They were preincubated at 37 °C for 2 h in the presence of 10.0 wt % low-molecular-weight PEGs of 200, 400, and 600 Da in DMEM before cryopreservation at −196 °C for 7 d. A significant difference in cell recovery rate was observed, with the best results in the small size SCSs preincubated in PEG200. Low-molecular-weight PEGs were not cytotoxic, and a significant difference in PEG population density in SCSs was well correlated with their recovery rate from cryopreservation. Not only recovery rate but also the health state of the SCSs was affected by the size of SCSs. The fusibility, proliferation, and differentiation activities were excellent for small SCSs preincubated in the presence of PEG200. The cryopreservation at −196 °C for 1 month maintained cell recovery rate >60% for small SCSs using the PEG200 cryoprotectant. This study suggests that the optimal size of SCSs, as well as the molecular weight of PEGs (cryoprotectant), is very important not only to enhance their high recovery rate from cryopreservation but also to maintain their bioactivities after cryopreservation.

## Ethical Approval

All procedures involving animals were conducted in adherence to the highest ethical standards. The Institutional Animal Care and Use Committees (IACUC) of Ewha Womans University provided approval for all animal-related protocols. These experimental procedures were executed in accordance with the guidelines laid out by the National Research Council.

## Data Availability

Data that support the findings of this study can be provided by the corresponding authors.
